# Dr. C. C. Johnson

**Published:** 1898-07

**Authors:** 


Dr. C. C. Johnson, of Gowanda, N. Y., died at his residence in that village, June 7, 1898. The members of the Lake Erie medical society attended the funeral, June 9th, in a body.

